# *Salmonella* and *S. aureus* Escape From the Clearance of Macrophages via Controlling TFEB

**DOI:** 10.3389/fmicb.2020.573844

**Published:** 2020-11-26

**Authors:** Shanshan Rao, Tao Xu, Yu Xia, Hongfeng Zhang

**Affiliations:** ^1^Department of Pathology, Wuhan Central Hospital, Huazhong University of Science and Technology, Wuhan, China; ^2^Cancer Biology Research Center, Tongji Hospital, Huazhong University of Science and Technology, Wuhan, China

**Keywords:** *Salmonella*, *S. aureus*, lysosome, TFEB, escape

## Abstract

Phagosome- and xenophagosome-lysosome systems play a critical role in the defense of pathogenic bacteria, such as *Salmonella* and *S. aureus*, in macrophages. A great part of the bacteria escapes from the digestion and can survive through some mechanisms that are still poorly understood and which require further exploration. Here we identified that *Salmonella* inhibited the expression and activation of TFEB to blunt the functions of lysosomes and defense of clearance by activating caspase-1. The expression and activation of TFEB were enhanced early under the infection of *S. aureus*, which was followed by shrinkage to weaken lysosomal functions due to the delayed activation of ERK, mTOR, and STAT3. Thus, we have identified novel escape mechanisms for *Salmonella* and *S. aureus* to deepen and strengthen our strategies fighting with pathogens.

## Introduction

Microbes have been alive in the world for thousands of years along with human beings, but the interplay between microbes and humans is still ongoing. When malignant bacteria infect the human body, the human immune system will work as a fighter against the invasion. The macrophage, as one of the innate immune cells, lends itself to protect us from bacterial infection first and foremost. In macrophages, phagosomes could act as a defense against a huge group of intracellular bacteria by fusing with lysosomes, but also a large group of invaders still survive through their processes of escape (Huang and Brumell, 2014). Meanwhile, the selective macro-autophagosome, known as xenophagosome, will capture the invaders or survivors to transport into lysosomes for clearance (Bauckman et al., 2015; Upadhyay and Philips, 2019; Xu et al., 2019). Obviously, lysosomes act as the terminator to suppress the survival of bacteria in macrophages.

The lysosome, known as “recycling center” of a cell, contains about 60 kinds of hydrolases within its single-membrane vesicles. Those hydrolases, such as cathepsins, collagenase, DNase, esterases, and so on work in an acidic pH (4.5–5.5) environment to degrade macromolecules, including lipid acids, DNA, and proteins (Perera and Zoncu, 2016; Ballabio and Bonifacino, 2020). Because of its powerful ability of digestion, disorders of lysosomal degradation result in a series of diseases, such as cancer, neurodegenerative diseases, metabolic disorders and so on, which are viewed as lysosomal storage disease (LSD) (Platt et al., 2018; Ballabio and Bonifacino, 2020). Specifically, inflammation and microbes infection are closely associated with lysosomes (Bauckman et al., 2015; Upadhyay and Philips, 2019; Xia et al., 2019; Xu et al., 2019).

TFEB, working as a critical regulator of autophagic and lysosomal functions, regulates the transcription of lysosomal membrane proteins and hydrolases, such as LAMP1/2, V-ATPase, cathepsin A/B/F, and so on (Settembre et al., 2011; Napolitano and Ballabio, 2016). The nuclear TFEB could bind to the 10-base E-box-like palindromic sequence (CLEAR) which is shared by the promoter sequence of lysosomal genes (Palmieri et al., 2011). While a group of protein kinases such as mTORC1, ERK2, and AKT could phosphorylate TFEB at ser142 or ser211, and the phosphorylated TFEB can be assembled to a 14-3-3 complex in cytoplasm (Settembre et al., 2011; Martina et al., 2012; Palmieri et al., 2017). When TFEB is dephosphorylated by calcineurin and released from the 14-3-3 complex, it will enter into nucleus for its transcriptional functions (Medina et al., 2015). Meanwhile, the expression level of TFEB also greatly contributes to its activities and functions.

Although it is well-studied that phagocytosis and xenophagy play a great role in the defense of bacteria, it is true that a lot of intracellular invaders survive and replicate (Huang and Brumell, 2014). Do *Salmonella* and *Staphylococcus aureus* (*S. aureus*), as two of common pathogenic bacteria, cross-talk with lysosomes? As reported, the process of phagocytosis could promote the activities of lysosomes, because pathogens bind to the Fcγ receptors and fuel the TFEB (Gray et al., 2016). *S. aureus* could activate TFEB to up-regulate the expression of inflammatory cytokines in RAW264.7 cells (Visvikis et al., 2014). Enhancing the activities of TFEB to restore the process of xenophagy to restrict the survival of *Salmonella* (Ammanathan et al., 2019). Yet, the detailed and critical mechanisms of how *Salmonella* and *S. aureus* regulate the functions of lysosomes and TFEB expression remain to be explored.

Here, we focused on how *Salmonella* and *S. aureus* regulated the activities of lysosomes and expression of TFEB through directly infecting bone marrow derived macrophages (BMDMs). We identified different regulating phenotypes that *Salmonella* down-regulated the critical genes of lysosomes and *Tfeb* striking as the activation of caspase1, while *S. aureus* enhanced the expression of TFEB early, and was reined in later because of the activation of ERK, mTOR, and STAT3 signals. If we inhibited the activation of caspase1 or ERK, mTOR, and STAT3 signals, BMDMs restored the full expression of *tfeb* and restrained the replication of *Salmonella* and *S. aureus*. Thus, we found two novel survival mechanisms of *Salmonella* and *S. aureus*.

## Results

### *Salmonella* Restricts the Expression of TFEB and Lysosomal Proteins, While *S. aureus* Boosts TFEB Early and Is Reined in Later

**FIGURE 1:**
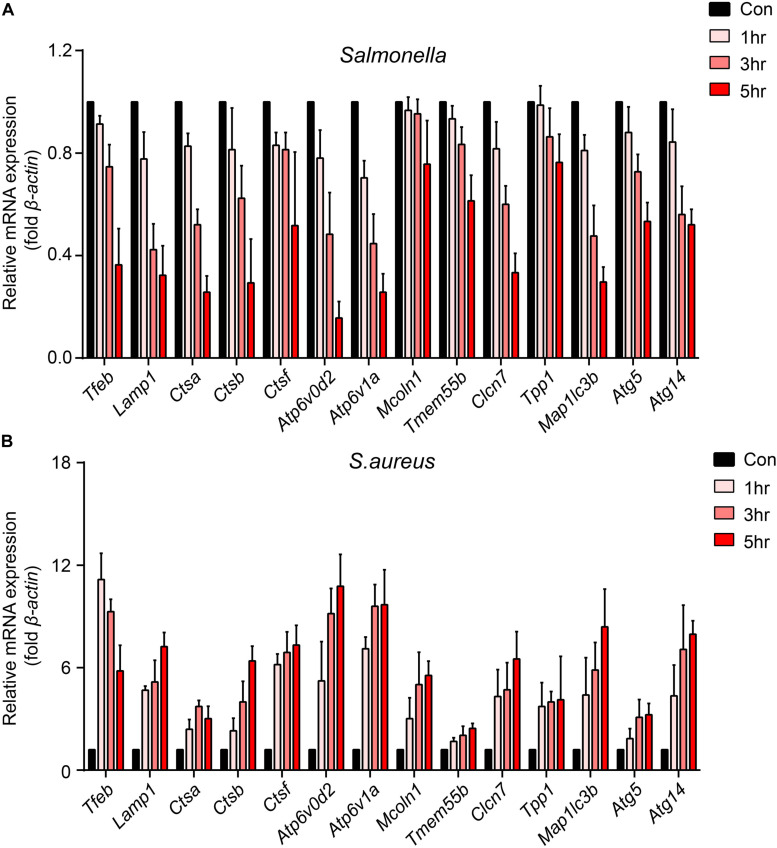
*Salmonella* inhibits the transcription of *Tfeb* and autophagosome-lysosome relative genes, while *S. aureus* enhances the transcription of *Tfeb* early and reins in later. **(A,B)** BMDMs were infected with *Salmonella* **(A)** or *S. aureus* **(B)** at a MOI of 5 for 0, 1, 3, 5 h and measured the level of genes with real-time PCR. The presented mean values (± SEM) were from at least three independent experiments **(A,B)**.

To explore how bacteria regulate the processes of lysosomal degradation, we infected BMDMs with *Salmonella* and *S. aureus* directly at a time gradient, and tested the lysosomal hydrolase genes, membrane genes and autophagic genes. Showing with histograms, we found that *Salmonella* remarkably restrained *Tfeb*, *Lamp1*, *V-Atpase*, hydrolase genes and autophagic genes, while *S. aureus* enhanced the transcription of those genes lightly (Figures 1A,B). Furthermore, under the infection of *S. aureus*, the level of *Tfeb* was up-regulated at 1 h, but gradually shrunk at 3 and 5 h (Figure 1B). As mentioned, *Tfeb* is an important transcription factor of lysosomal and autophagic genes, and thus we supposed that *Salmonella* and *S. aureus* controlled the transcription of *Tfeb*, which resulted in regulation of lysosomal and autophagic genes.

**FIGURE 2:**
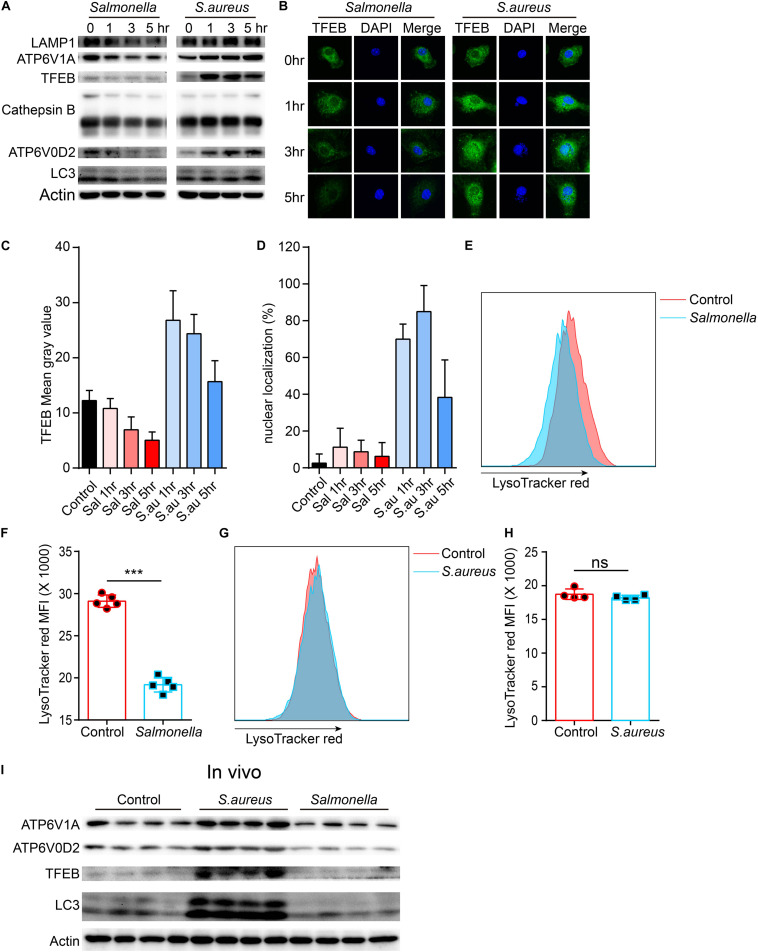
*Salmonella* restrains the expression of TFEB and lysosomal proteins, while *S. aureus* boosts the expression of TFEB early and reins back later. **(A,B)** BMDMs were infected with *Salmonella* or *S. aureus* at a MOI of 5 for 0, 1, 3, 5 h. To measure the level of proteins with western-blot **(A)** and stain TFEB or nucleus with anti-TFEB antibody or DAPI **(B)**. **(C,D)** Quantification of the level of TFEB in cells at least five views **(C)** and the percentage of nuclear TFEB per cell (6 cells) in each group with Image J **(D)**. **(E–H)** BMDMs were infected with *Salmonella* or *S. aureus* for 5 h, and stained with LysoTracker red for 10 min, the representative images of MFI are shown **(E,G)**, and the quantitative data are shown **(F,H)**. Histograms depict mean values (± SEM). ***p < 0.001. **(I)** Mice were infected with *Salmonella* or *S. aureus* for 8 h and the level of TFEB in peritoneal macrophages was measured with western-blot (*n* = 4 in each group). Representative bands and pictures were from three independent experiments **(A,B,E,G)**.

To confirm this, we tested the protein level of lysosomes and LC3 upon the infection of the two bacteria. The TFEB, LAMP1, ATP6V1A, ATP6V0D2, cathepsin B and LC3 were strongly inhibited by *Salmonella*. It was same with this gene that the protein level of TFEB was first enhanced, when BMDMs were cocultured with *S. aureus*, and then reined back (Figure 2A and Supplementary Figures S1A–F). We also verified with Immunofluorescence, and found similar phenotypes (Figures 2B,C). Meanwhile, nuclear localization rates of TFEB were notably increased at 1, 3 h and declined at 5 h with the infection of *S. aureus*, but not obviously in *Salmonella* group (Figures 2B,D). This means a discrepant regulation of TFEB activity by the two bacteria. Furthermore, we used LysoTracker red to stain acid vacuoles, and the mean fluorescence intensities (MFI) might relatively represent the lysosomal acidic strength. After infection for 5 h, we found that *Salmonella* could weaken the lysosomal acidity, while *S. aureus* could not (Figures 2E–H). Within vivo, C57/B6 mice were infected with *S. aureus* or *Salmonella* via intraperitoneal injection, and then we collected the peritoneal macrophages for testing with western-blot. We found TFEB, ATP6V1A, ATP6V0D2, and LC3 were increased under the treatment of *S. aureus* and inhibited by *Salmonella* (Figure 2I and Supplementary Figure S1G).

### *S. aureus* Strikingly Activates ERK, mTOR, NFκB, and STAT3 Signaling Pathways, While *Salmonella* Activates Caspase-1

**FIGURE 3:**
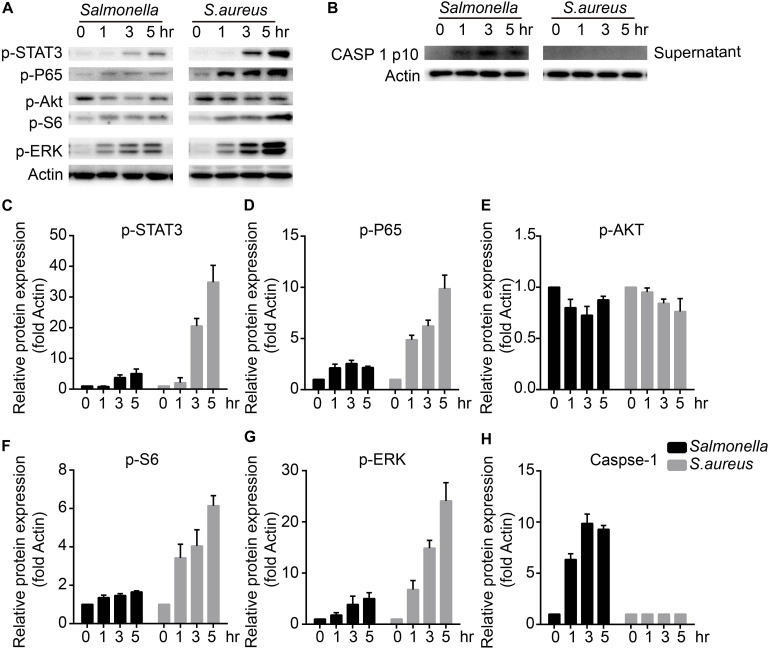
*S. aureus* activates ERK, mTORC, NFκB and STAT3 signaling pathways, while *Salmonella* activates caspase-1. **(A–H)** BMDMs were infected with *Salmonella* or *S. aureus* at a MOI of 5 for 0, 1, 3, 5 h. To measure the activation of STAT3, P65, AKT, S6, ERK and caspase-1 released in the culture medium with western-blot **(A,B)**, and histograms show the quantitative statistics of those enzymes and caspase p10 level **(B–H)**. Representative bands were from three independent experiments **(A,B)**.

To figure out the detailed mechanisms, we infected BMDMs with *S. aureus* or *Salmonella* for 0, 1, 3, 5 h, and checked several signaling pathways that might regulate the expression and activity of TFEB. We found that ERK, mTOR, NFκB, and STAT3 could be activated obviously with treatment of *S. aureus*, but only slightly activated by *Salmonella* (Figures 3A,C–G). Meanwhile, as reported, The bacterium such as *Salmonella* or *Listeria* could activate inflammasomes (Wu et al., 2010; Zhao et al., 2011; Qu et al., 2016), and we tested the mature-caspase 1 level that was released to the supernatant. The results were that *S. aureus* could not activate caspase-1 but *Salmonella* could (Figures 3B,H).

### VX-765 Restores the Expression of TFEB and Lysosomal Proteins With Administration of *Salmonella*, While ERKi, Rapamycin and S31-201 Accelerate the Expression of TFEB and Lysosomal Proteins With Administration of *S. aureus*

**FIGURE 4:**
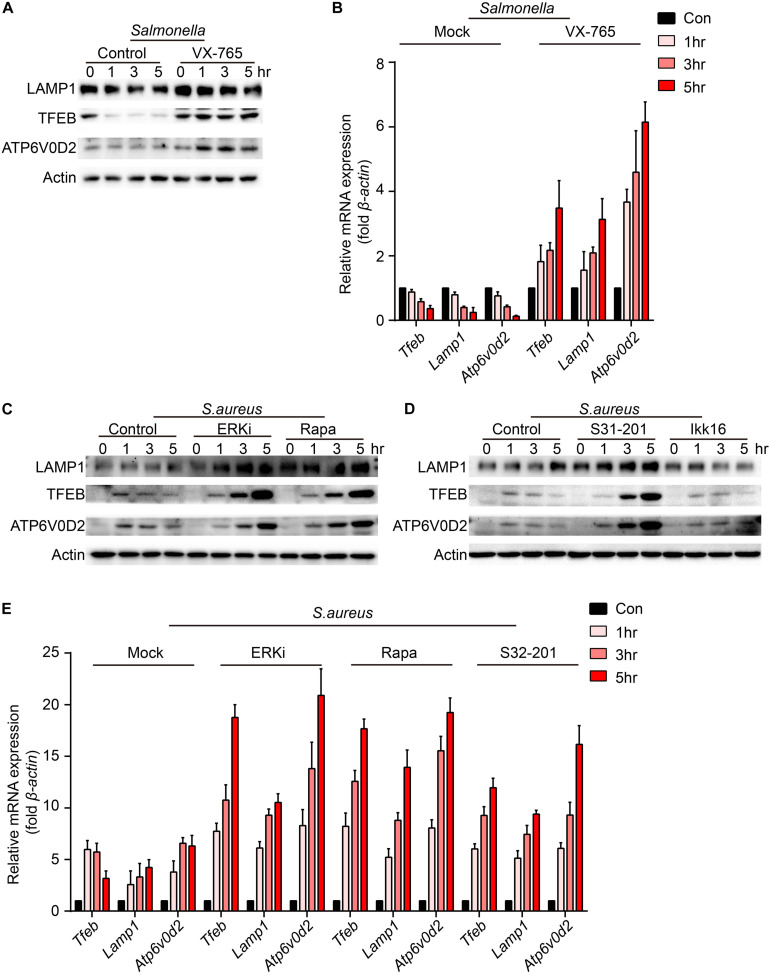
VX-765 restores the level of TFEB and lysosomal proteins with infection of *Salmonella*, while ERKi, rapamycin and S31-201 accelerate the expression of TFEB and lysosomal proteins with infection of *S. aureus*. **(A,B)** BMDMs were pre-treated with 5 μM VX-765 for 1 h and then infected with *Salmonella* at an MOI of 5 for 0, 1, 3, 5 h. Testing the protein level **(A)** and gene level **(B)** of TFEB, LAMP1 and ATP6V0D2. **(C–E)** BMDMs were pre-treated with 500 nM SCH772984, 100 nM rapamycin, 10 μm S31-201, 200 nM IKK 16 or DMSO for 1 h and then infected with *S. aureus* at a MOI of 5 for 0, 1, 3, 5 h. Testing the protein level **(C,D)** and gene level **(E)** of TFEB, LAMP1 and ATP6V0D2. Representative bands were from three independent experiments **(A,C,D)** and the presented values of means (± SEM) were from three independent experiments **(B,E)**.

To verify which signal could regulate the expression of TFEB and lysosomal proteins, BMDMs were pre-treated with caspase-1 inhibitor, ERKi, rapamycin, S31-201 or IKK16 upon the infection of *Salmonella*. Because LAMP1 is a key marker of lysosome and ATP6V0D2 has a great role in the autophagy-lysosome degradation process (Xia et al., 2019), we tested the protein and gene level of the two and TFEB. We found that TFEB, LAMP1, and ATP6V0D2 did not decrease anymore with treatment of VX-765 (Figures 4A,B and Supplementary Figure S2A), while there were no significant changes under the administration of ERK, mTOR, STAT3, or NFκB inhibitor (Supplementary Figures S2B,C). When BMDMs were infected with *S. aureus*, the LAMP1 and ATP6V0D2 were much more obviously increased and TFEB was continuously up-regulated in a time-dependent manner after treatments with ERK, mTOR and STAT3 inhibitor (Figures 4C–E and Supplementary Figure S3A). Meanwhile, BMDMs were treated with those inhibitors alone that could induce non-significant responses (Supplementary Figures S4A–D). Together, we suggested that *Salmonella* could inhibit the expression of TFEB through the activation of caspase-1, and the detailed mechanisms need further follow-up investigations. While *S. aureus* could block the sufficient expression of TFEB and lysosomal genes by activating ERK, mTOR, STAT3.

### VX-765 Restores the Activity of TFEB With Administration of *Salmonella*, While ERKi, Rapamycin and S31-201 Promote the Activity of TFEB Under Infection of *S. aureus*

**FIGURE 5:**
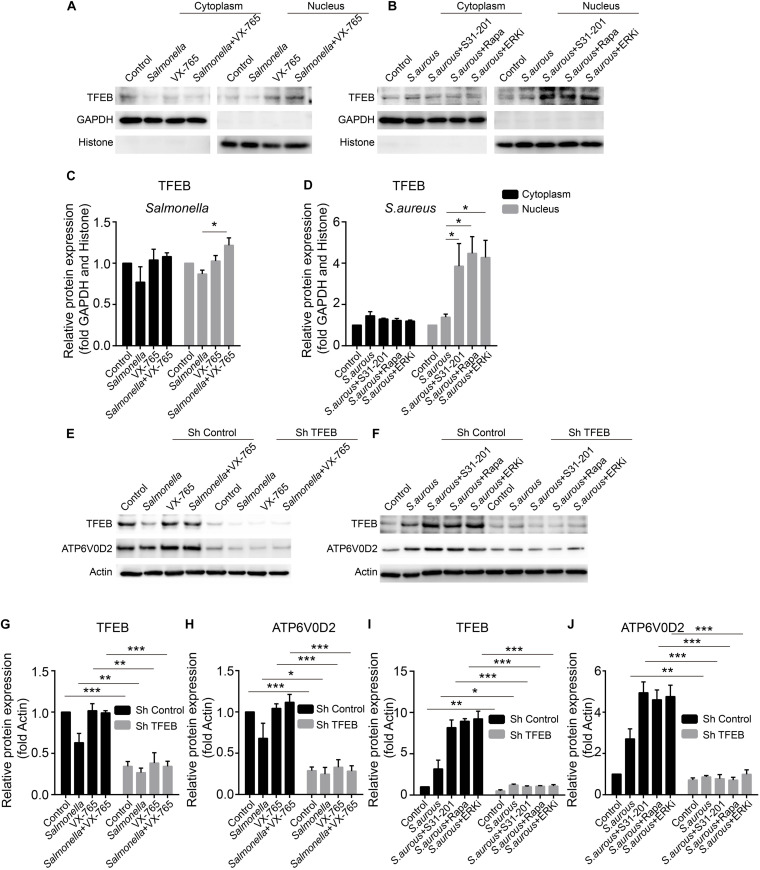
VX-765 restores the activity of TFEB with infection of *Salmonella*, while ERKi, rapamycin and S31-201 promote the activity of TFEB under infection of *S. aureus*. **(A,B)** Mature BMDMs were plated into 6-well plates, and infected with *Salmonella* and *S. aureus* alone or combined with inhibitors for 3 h, and then, the cytoplasmic and nuclear TFEB were tested with western-blot. **(C,D)** The histograms show the statistics of distribution of TFEB in cytoplasm and nucleus. **(E–J)** Using lentivirus to knock down TFEB in BMDMs, and those cells were treated with bacteria alone or combined with inhibitors for 3 h. TFEB and ATP6V0D2 were tested with western-blot **(E,H)** and the quantitative data were displayed by histograms **(G–J)**. Histograms depict mean values (± SEM). *p < 0.05; **p < 0.01; ***p < 0.001. Representative bands were from three independent experiments **(A,B,E,F)**.

The expression level of TFEB was positive-regulated by the inhibitors upon the infection of *Salmonella* and *S. aureus*. To explore the activity of TFEB under bacterial infection with the treatment of inhibitors, we isolated the cytoplasm and nucleus of BMDMs to check the level of TFEB. The results showed that VX-765 could promote the TFEB to transfer into the nucleus under infection of *Salmonella* (Figures 5A,C), while S31-201, rapamycin and ERKi could dramatically increase the nuclear TFEB upon infection of *S. aureus* (Figures 5B,D). Meanwhile, to further verify the role of TFEB in the regulation of lysosomal proteins under bacterial infection, we constructed lentivirus to knock down *Tfeb* in BMDMs, and then infected with *Salmonella* and *S. aureus*. We found that if TFEB was knocked down, the ATP6V0D2 was obviously down-regulated on infection of bacteria alone or combined with inhibitors (Figures 5E–J).

### VX-765 Reconstitutes the Defense of *Salmonella*, While ERKi, Rapamycin and S31-201 Enhance the Defense of *S. aureus*

**FIGURE 6:**
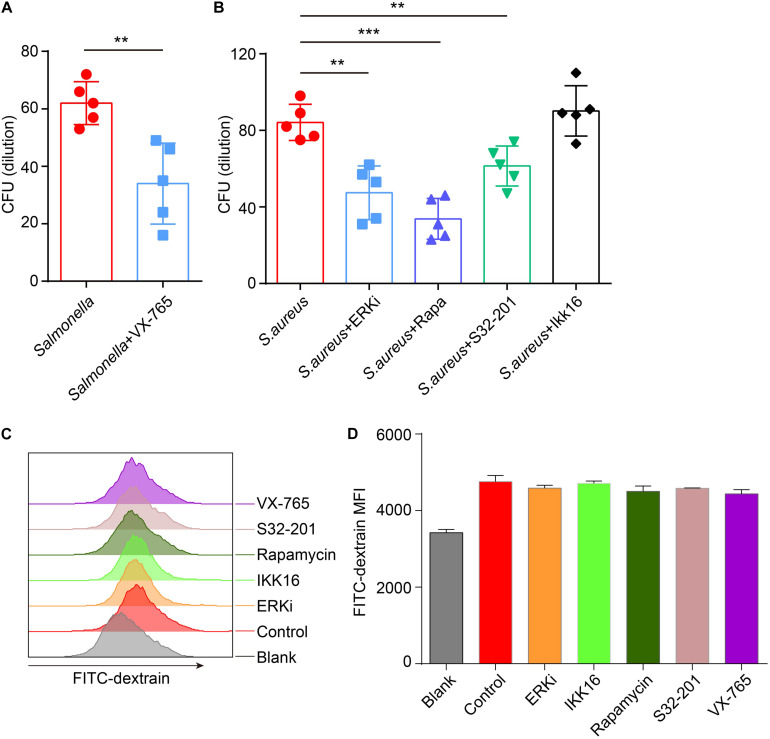
VX-765 reconstitutes the defense of *Salmonella*, while SCH772984, rapamycin and S31-201 enhance the defense of *S. aureus*. **(A,B)** 5 μM VX-765-primed BMDMs were infected with *Salmonella* at a MOI of 2 **(A)**, and 500 nM SCH772984-, 100 nM rapamycin-, 10 μm S31-201- or 200 nM IKK 16-primed BMDMs were infected with *S. aureus* at a MOI of 2 **(B)**. Checking the survived bacteria per cell with gentamicin protection assay. **(C,D)** Inhibitor-primed BMDMs were incubated with FITC-Dextrain for 1 h and the MFI of FITC was measured with Flow cytometer **(C)**, and the MFI quantified **(D)**. Histograms depict mean values (± SEM). **p < 0.01; ***p < 0.001. The presented values of means were from at least three independent experiments.

Next, to confirm if the defense of bacteria could be reconstituted or enhanced after administration with inhibitors, inhibitors-primed BMDMs were cocultured with *Salmonella* or *S. aureus*, and measured the number of survivors in BMDMs with a gentamicin protection assay. The results announced that with treatment of caspase-1 inhibitor, less *Salmonella* survived than in the control group (Figure 6A), and less *S. aureus* were alive after administration of ERK, mTOR and STAT3 inhibitor (Figure 6B). To exclude the different endocytosis treatment with inhibitors, inhibitor-primed BMDMs were cocultured with FITC-Dextrain, and the results showed that there were non-significant changes among those inhibitor treatments (Figures 6C,D). Thus, with reconstituting or promoting the expression of TFEB by caspase-1 inhibitor or ERK, mTOR and STAT3 inhibitors, BMDMs strengthened the defense against the invasion of *Salmonella* and *S. aureus*.

## Discussion

Phagosome-lysosome and autophagosome-lysosome, as two critical mechanisms of digestion defense against the invasion of pathogens, could clear most intruders. But there are a large group of bacteria that continue to survive under excessive infection. Meanwhile, these pathogens have evolved some escape approaches to resist the clearance of immune cells (Huang and Brumell, 2014). In this study, we examined novel escape mechanisms for *Salmonella* and *S. aureus* with controlling the expression and activity of TFEB, and then regulating the degradation function of lysosomes. We also verified the detailed signaling pathways that participated in the regulation of the expression and function of TFEB and lysosomes, and through blocking those pathways restored or enhanced the function of lysosomes and defense of bacteria.

*Salmonella* is a classical intracellular pathogenic germ which induces colitis and poses a huge health burden (Hartz et al., 1950; Herp et al., 2019). Several escape mechanisms have been found that are used by *Salmonella* in order to survive. For example, when they infect epithelial cells or macrophages, those intracellular bacteria are wrapped up in vacuoles, termed as *Salmonella*-containing vacuoles (SCVs), through its T3SSs (SPI-1 and SPI-2) for better replication and ability to survive (Birmingham et al., 2006). Meanwhile, at the early invasion, *Salmonella* could also trigger xenophagy as consuming energy and amino acid, but the process would quickly be suppressed via degrading Sirt1/LKB1/AMPK complexes (Ganesan et al., 2017). However, the detailed processes for escape should be further explored.

Under the infection of *Salmonella*, NLRC4 and NLRP3 inflammasomes could be activated within 1 h, cut the pro-caspase-1 into active caspase-1 in macrophages and induce pyroptosis (Monack et al., 2000; Lara-Tejero et al., 2006; Zhao et al., 2011; Qu et al., 2016). As reported, the totally knock-out *Caspase1* in mice, *Caspase1*^–/–^ mice, even increased the susceptibility to *Salmonella* infection *in vivo* via other mechanisms, such as deficiency of cytokines or involvement of neutrophils (Lara-Tejero et al., 2006; Raupach et al., 2006; Miao et al., 2010). Although Denise M. Monack’s group had published that intracellular living bacteria were equal between WT and *Caspase1*^–/–^ macrophages through gentamicin protection assay (Monack et al., 2000), the caspase1-depdent cell death was robustly decreased in the caspase1^–/–^ group (Lara-Tejero et al., 2006). Thus, whether or not caspase1 is involved in the defense of *Salmonella* in macrophages also needs to be investigated. In our present study, we had identified a critical role of caspase1 in regulating the expression of TFEB, and TFEB contributed to the resistance of pathogens involving *Salmonella* (Huang and Brumell, 2014; Ammanathan et al., 2019). In our gentamicin protection assay, we measured the living bacteria in each well by folding β-actin, and inhibition of caspase-1 activity obviously stunted the replication of *Salmonella*. Therefore, we introduced a new surviving pathway, “*Salmonella*-caspase1-TFEB-lysosome digestion-*Salmonella* survive,” for *Salmonella*.

*S. aureus* is a common pathogenic bacteria to induce systemic infections or abscesses for humans and animals, although it is commensal most of the time. Not only do neutrophils act as the first responders under the infection of *S. aureus*, but also macrophages have a critical role in clearance of the bacteria (Koziel et al., 2009; Flannagan et al., 2016; Moldovan and Fraunholz, 2019). The professional phagocytes, macrophages, uptake *S. aureus* within minutes to capture the bacteria with Rab-5 positive phagosomes, and those phagosomes need further maturation. Mature phagosomes can be fused with lysosomes to form phagolysosomes for killing bacteria (Moldovan and Fraunholz, 2019). But, a great part of those phagolysosomes could not completely digest *S. aureus* as they are lacking some necessary hydrolases (Jubrail et al., 2016). Those survivors could replicate in the acidic vacuoles and finally cause macrophage death (Jubrail et al., 2016; Tranchemontagne et al., 2016). Hence, we focused on the critical mechanisms for the escape of *S. aureus* in macrophages. Here, we found that *S. aureus* could activate some key protein kinases, such as ERK, mTOR, STAT3 and so on. ERK and mTOR had been well-studied to control the activity and expression of TFEB, while STAT3 was first verified to blunt the function of TFEB in macrophages under *S. aureus* infection (Li et al., 2018). Insufficient TFEB led to the disabling lysosomes and more bacteria survived.

In summary, in the battle of bacteria and macrophages, bacteria have generated some escape pathways for better survival. We have verified the great role of TFEB in the defense of bacteria, and explored critical mechanisms to regulate the expression and activity of TFEB during the infection of *Salmonella* and *S. aureus*. Those discoveries contribute to the strategies for the cure of infection dramatically.

## Materials and Methods

### Reagents

Anti-LAMP1 (sc-20011), anti-caspase1 p10 (sc-514) antibodies were purchased from Santa Cruz Biotechnology. Anti-TFEB (ab2636), anti-ATP6V1A (ab 137574), donkey anti-goat IgG H&L (FITC; ab6881) antibodies were from Abcam. Anti-ATP6V0D2 (SAB2103221) antibody, DAPI (d9542) came from Sigma Aldrich. Anti-cathepsin B (#31718), anti-LC3B (#3868), anti-actin (#3700), phospho-STAT3 (Tyr705; #9145), phospho-NF-κB P65 (Ser536; #3033), phospho-AKT (Ser473; #4060), phospho-P44/42 MAPK (ERK1/2; Thr202/Tyr204; #4370), and-GAPDH (5174S) antibodies were purchased from Cell Signaling Technology. Anti-Histone H3 (ab1791) antibody was purchased from Abcam. SCH772984 (ERKi; S7101), rapamycin (mTORCi; S1039), S3I-201 (STAT3i; S1155), IKK-16 (IKK Inhibitor VII; S2882), VX-765 (caspase1i; S2228) were from Selleck. Gentamicin sulfate (1289003) and FITC-Dextran (FD4) were from Sigma Aldrich. LysoTracker red NDN-99 (L7528) was purchased from Thermo Fisher Scientific.

### Cell Culture and Stimulation

All experiments were performed *in vitro* with mouse primary macrophages that were derived from bone marrow cells (BMDMs). Those cells were cultured with DMEM (Thermo Fisher Scientific, 11965092) medium containing 10% FBS, penicillin, streptomycin and 50ng/ml M-CSF (PeproTech, 315-02) for 7 days. BMDMs were cocultured with *Salmonella* and *S. aureus* at a desired MOI (2:1) in the gentamicin protection assay, while MOI (5:1) in direct infection or inhibitor-primed infection experiments.

### mRNA Isolation and Real-Time PCR

Total mRNA was extracted from infected BMDMs with TRI Reagent (Sigma Aldrich; 93289) and reverse transcription using a kit from Thermo Fisher Scientific (4374966). Real-time PCR was performed with SYBR^TM^ Green mix (Thermo Fisher Scientific, A25742). All real-time PCR primers were listed as follows,

*Tfeb*-F: TTCTGCCCGGACTCAGTTTC;

*Tfeb*-R: TCTCGGGGTTGGAGCTGATA;

*Lamp1*-F: GCCTCAGCACTCTTTGAGGT;

*Lamp1*-R: GTTGGGGAAGGTCCATCCTG;

*Atp6v0d2*-F: TGCGGCAGGCTCTATCCAGAGG;

*Atp6v0d2*-R: CCACTGCCACCGACAGCGTC;

*Ctsb*-F: GGCCCAGTGGAGGGTGCCTT;

*Ctsb*-R: TGCGTGGGATTCCAGCCACAA;

*Ctsf*-F: CCACCTTGCAATGATCCCCT;

*Ctsf*-R: TTCACTGGGCTACAGTCCCT;

Ctsa-F: GGAGAGCAAGGACGCAAGG;

Ctsa-R: TGGCAATCAGGTTCCAAGCA;

*Mcoln1*-F: TTGCTCTCTGCCAGCGGTACTA;

*Mcoln1*-R: GCAGTCAGTAACCACCATCGGA;

*Tmem55b*-F: GTTCGATGCCCCTGTAACTGTC;

*Tmem55b*-R: CCCAGGTTGATGATTCTTTTGC;

*Clcn7*-F: GAGGAGGGACCTCAGTCTCA;

*Clcn7*-R: GGAGCTTCTCGTTGTGTGGA;

*Tpp1*-F: ATCTGGAACCTCGGCCTCTA;

*Tpp1*-R: CCTGTCCCATGCTGCTGATA;

*Map1lc3a*-F: TTGGTCAAGATCATCCGGCG;

*Map1lc3a*-R: TCTTGGGAGGCGTAGACCAT;

*Atg5*-F: TGCATCAAGTTCAGCTCTTCCT;

*Atg5*-R: CTGGGTAGCTCAGATGCTCG;

*Atg14*-F: GCTTCGAAGGTCACACATCC;

*Atg14*-R: CTTGAGGTCATGGCACTGTC;

### Scrambled shRNA Lentiviral Plasmid Construction and Lentiviral Particles Generation

A short hairpin RNAs pair which targets *Tfeb* was cloned into pLVX-shRNA2 plasmid (Clontech Laboratories, Inc. 632179) with BamH I and EcoR I according to the protocol of ClonExpress^®^ Ultra One Step Cloning Kit (C115) purchased from Vazyme, and the shRNA sequence as follow, forward: 5′-GATC CGGCAGTACTATGACTATGATTTCAAGAGAATCATAGTCA TAGTACTGCCGTTTTTG-3′, reverse: 5′-AATTCAAAAACGG CAGTACTATGACTATGATTCTCTTGAAATCATAGTCATAG TACTGCCG-3′. For producing lentiviral particles, the vector and packaging plasmids were transfected into 293T cells about 36 h and harvested the supernatant which contained lentivirus. Using these virus particles to transfect BMDMs to knock down *Tfeb.*

### Western-Blot

Cellular proteins were extracted with RIPA buffer containing proteases and phosphatase inhibitor cocktail (Thermo Fisher Scientific, 78440) and quantified with a BCA kit (Thermo Fisher Scientific, A53226). Then, protein lysis was mixed with loading buffer and boiled at 100°C for 5–10 min. The well-prepared lysis was loaded into 10% SDS-PAGE gel for electrophoresis and transferred on to a PVDF membrane. Before being incubated with antibodies against TFEB, LAMP1, ATP6V0D2, ATP6V1A, cathepsin B, LC3, p-STAT3, p-P65, p-PS6, p-ERK, p-AKT, caspase1 p10, and actin, the membranes should be blocked with 5% BSA, and followed by incubating with HRP-secondary antibodies (Cell Signaling Technology, 7074; Abcam, ab6885). Finally, the membranes were exposed under X ray and the bands were quantified with Image J.

### Immunofluorescence Staining and Confocal Microscopy

The cells were seeded on glass slides and fixed with 4% formaldehyde for 30 min, permeabilized by 0.05% Triton X-100 about 20 min, blocked with 5% BSA for 1 h and incubated with anti-TFEB antibody overnight at 4°C. The cells were incubated with FITC-anti-goat secondary antibody for 1 h and DAPI for 10 min in darkness. After fully washing, the slides were placed onto confocal microscopy (Zeiss, Germany) for taking fluorescent photos.

### FITC-Dextran and LysoTracker Red Staining

The mature BMDMs were cultured in 48-well plates, and treated with 5 μM VX-765, 500 nM SCH772984, 100 nM rapamycin, 10 μm S31-201, 200 nM IKK 16 or DMSO for 1 h, and incubated with FITC-Dextran for 1 h, then the MFI was measured with a Flow cytometer (CytoFLEX, Beckman Coulter). For staining LysoTracker red, BMDMs were plated in 12-well plates, and cultured with *Salmonella* and *S. aureus* at 5 MOI for 5 h, and then, stained with LysoTracker red for 10 min. Lastly, the MFI was measured with a Flow cytometer (CytoFLEX, Beckman Coulter).

### Infection Model *in vivo* and Bacteria Culture

The C57/B6 mice were purchased from *shanghai model organisms*. And our animal model was constructed according to the guidelines of the Institutional Animal Care. *Salmonella* (SL1344) and *S. aureus* (NCTC8325) were a gift from Xiang-ping Yang Lab. In the infection model, the single colonies of *Salmonella* and *S. aureus* were shaken in 2–4 ml LB medium for 5 h at 37°C and the concentration of bacteria was quantified to reach an OD_600_ of 0.5 with a spectrophotometer (Thermo Fisher Scientific^TM^). Those sex-and age-matched mice were divided into three groups, including control group, *Salmonella* infection group, and *S. aureus* infection group. The experimental groups were administrated with 1 × 10^8^ bacteria suspended in 0.5 ml DMEM medium through intraperitoneal injection for 8 h, and then the mice were sacrificed. Five milliliter germ-free PBS was injected into the peritoneum of the mice and kneaded gently. The macrophages were harvested from the ascites, and the cells lysed with RIPA buffer for western-blot.

### Gentamicin Protection Assay

*Salmonella* and *S. aureus* were generated from single colonies as before. Mature BMDMs were cocultured with bacteria at an MOI of 2 for 1 h. After three rounds of washing with PBS, the infected BMDMs were incubated with 300 μg/ml gentamicin and 100 μg/ml gentamicin diluted in DMDM medium in turn for 1 h. After three rounds of washing with PBS, the BMDMs were lysed with 0.02% Triton x-100. Then one half of the lysis was diluted with LB into appropriate concentrations and seeded on LB plates for measuring the preliminary CFU. Another half of the protein lysis was quantified with a BCA kit and the final CFU per cell in each group was generated by the preliminary CFU folding protein concentration in each well.

### Statistics

All of our data were quantified and drawn with GraphPad Prism 5 and presented as means (± SEM). The measurement of those data through a two tailed Student’s *t*-test and *p*-values, considered as significant, should be less than 0.05.

## Data Availability Statement

The original contributions presented in the study are included in the article/Supplementary Material, further inquiries can be directed to the corresponding authors.

## Ethics Statement

The animal study was reviewed and approved by the Wuhan Central Hospital, Huazhong University of Science and Technology, Wuhan. Written informed consent was obtained from the owners for the participation of their animals in this study.

## Author Contributions

SR and YX conceived and performed the experiments and analyzed the data. SR, TX, YX, and HZ wrote the manuscript. YX and HZ supervised the project. All authors contributed to the article and approved the submitted version.

## Conflict of Interest

The authors declare that the research was conducted in the absence of any commercial or financial relationships that could be construed as a potential conflict of interest.
