# Supplementary material for: Salmonella and S. aureus Escape From the Clearance of Macrophages via Controlling TFEB
Source: Front Microbiol. 2020 Nov 26;11:573844. doi: 10.3389/fmicb.2020.573844 (PMC7726115; doi:10.3389/fmicb.2020.573844)

Salmonella and *s.aureus* escape from the clearance of macrophages via controlling TFEB

Shanshan Rao[1], Tao Xu[2], Yu Xia[2]* and Hongfeng Zhang[1]*

1, Department of Pathology, Wuhan Central Hospital, Huazhong University of Science and Technology, Wuhan, 430014, China;
2, Cancer Biology Research Center, Tongji Hospital, Huazhong University of Science and Technology, Wuhan, 430030, China

Figure S1. *Salmonella* restrains the expression of TFEB and the expression of lysosomal proteins, while *s.aureus* boosts the expression of TFEB early and decreases later. (A-F) Histograms show the statistics of LAMP1, ATP6V1A, TFEB, cathepsin B, ATP6V0D2, and LC3 in BMDMs under the treatment of *salmonella* and *s.aureus* at a time gradient. (G) Histogram shows the ATP6V1A, ATP6V0D2, TFEB and LC3 in peritoneal macrophages under the administration of *s.aureus, salmonella* or not in vivo.

Figure S2. VX-765 restores the expression level of TFEB and lysosomal proteins with infection of *salmonella*. (A) Histograms show the LAMP1, TFEB, and ATP6V0D2 in BMDMs under the treatment of *salmonella* or combined with VX-765. (B, C) BMDMs were infected with *salmonella* with 500 nM SCH772984, 100 nM

rapamycin, 10 μm S31-201 or 200 nM IKK 16 for 0, 1, 3, 5 hours, and LAMP1, TFEB, ATP6V0D2, actin were measured with western-blot (B), and quantified the expression level of those proteins (C). The representative bands were from three independent experiments (B).

Figure S3. ERKi, rapamycin and S31-201 accelerate the expression of TFEB and lysosomal proteins with infection of *s.aureus*. (A) Histograms show the expression level of LAMP1, TFEB and ATP6V0D2 under the treatment of *s.aureus* alone or combined with 500 nM SCH772984, 100 nM rapamycin, 10 μm S31-201 or 200 nM IKK 16 for 0, 1, 3, 5 hours.

Figure S4. VX-765, ERKi, rapamycin, S31-201 and IKK16 do not obviously up-regulate the expression of TFEB and lysosomal proteins by themselves. (A-D) mature BMDMs were treated with 5 μM VX-765, 500 nM SCH772984, 100 nM rapamycin, 10 μm S31-201 or 200 nM IKK 16 for 0, 1, 3, 5 hours. LAMP1, TFEB, ATP6V0D2, actin were measured with western-blot (A, C) and quantified (B, D). The representative bands were from three independent experiments (A, C).

Supplemental Figure 1

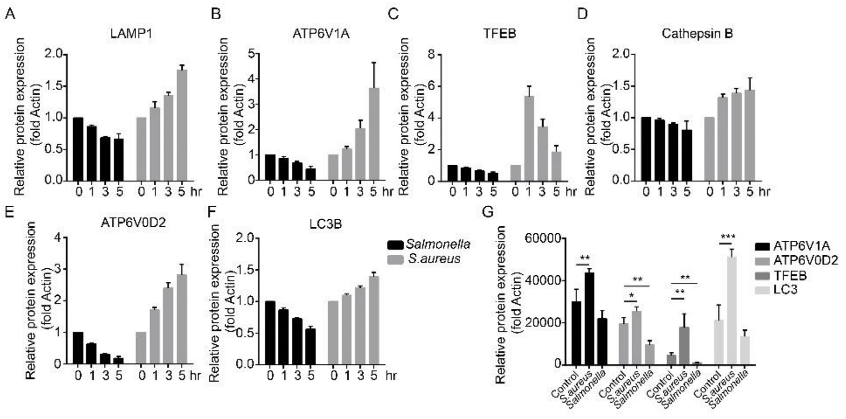

Supplemental Figure 2

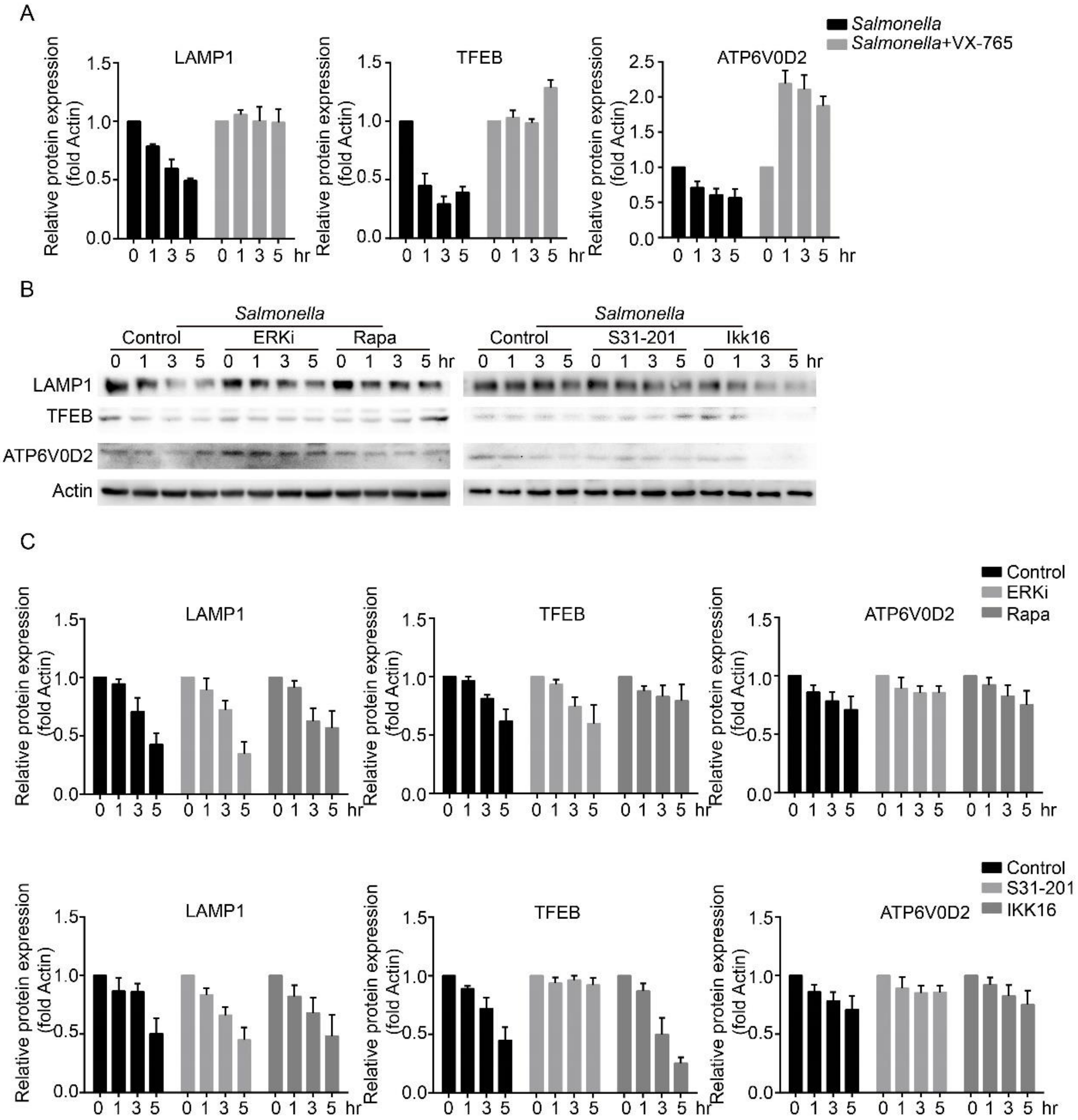

Supplemental Figure 3

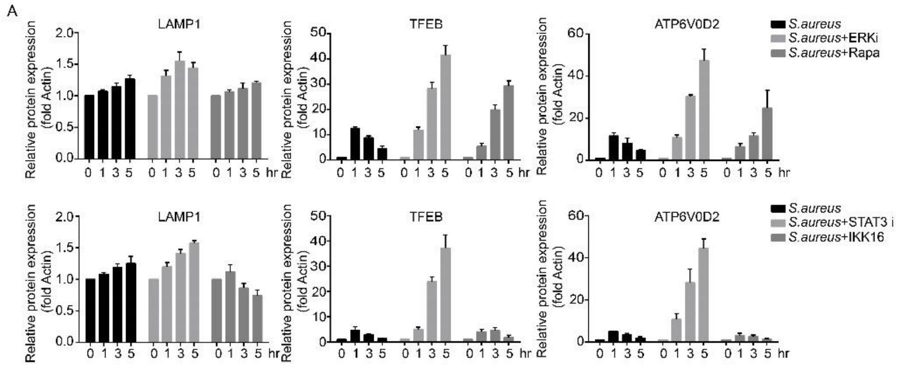

Supplemental Figure 4

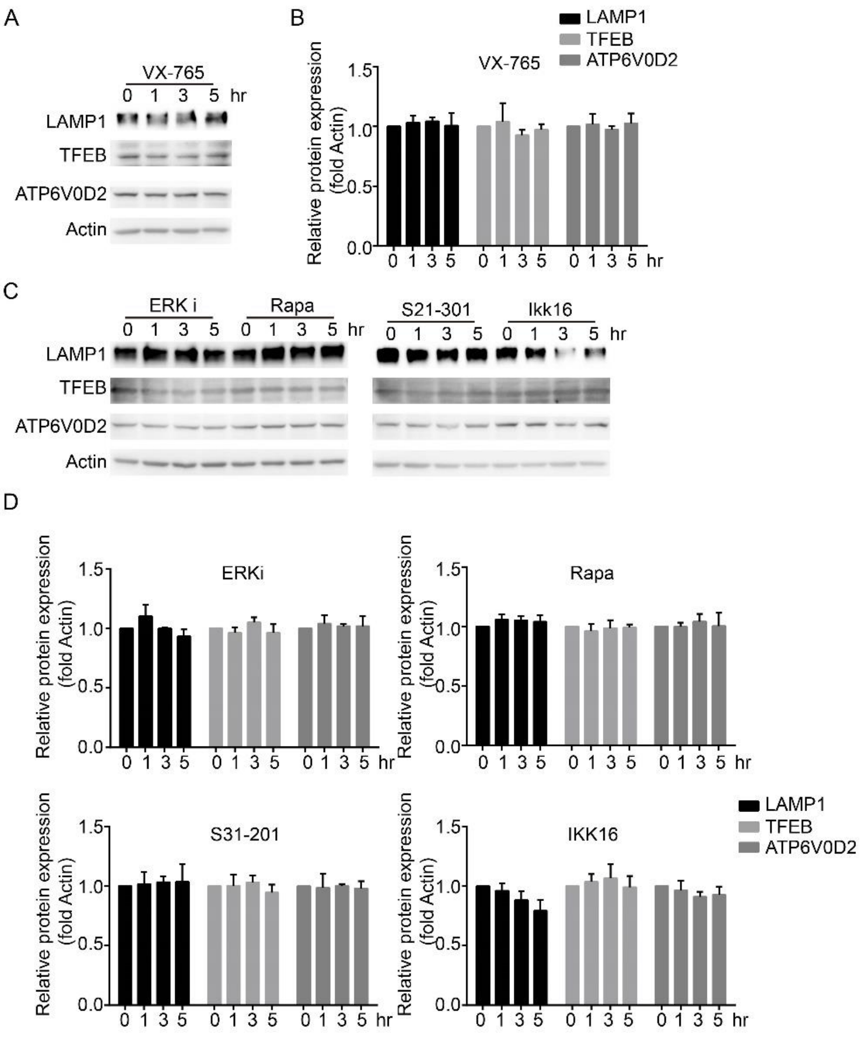

Supplement: Supplementary file 1 [file Data_Sheet_1.pdf]
